# Emerging risk of *Dirofilaria* spp. infection in shelter dogs in southern Italy

**DOI:** 10.3389/fvets.2023.1112036

**Published:** 2023-07-06

**Authors:** Lavinia Ciuca, Valeria Caruso, Sergio Illiano, Antonio Bosco, Maria Paola Maurelli, Laura Rinaldi

**Affiliations:** Department of Veterinary Medicine and Animal Production, Unit of Parasitology, University of Federico II, Naples, Italy

**Keywords:** *Dirofilaria immitis*, *Dirofilaria repens*, southern Italy, shelter dogs, zoonotic risk

## Abstract

In southern Italy, the number of autochthonous cases of *Dirofilaria immitis* in dogs has increased considerably. This also occurs in the Campania region, particularly in coastal areas, where infections with *D. immitis* and *Dirofilaria repens* have been reported more frequently. Therefore the aim of the present study was to better investigate the occurrence of *Dirofilaria* spp. in a local dog shelter in Castel Volturno (Campania region, southern Italy). Briefly, a total of 260 blood samples were analysed for identification of microfilariae (mff) and detection of *Dirofilaria immitis* antigen. Dogs were classified according to their age (1–3  years; 4–6  years; 7–11  years; > 11  years) and length of stay in the shelter at the time of sampling (dogs that entered in the shelter in the last 4  months; dogs housed in the shelter for more than 4 months up to 2  years; dogs housed for more than 2  years). The modified Knott’s test revealed that 195 dogs (75.0%) were positive for circulating mff of *Dirofilaria* spp. Specifically, 104/260 (40.0%) dogs were positive for *D. immitis* and 91/260 (35.0%) were positive for *D. repens*. In addition, 72/260 (27.7%) dogs had both *D. immitis* and *D. repens* mff. Antigen testing revealed that 78/260 (30.0%) dogs were positive for *D. immitis*. However, 26/104 (25.0%) of the dogs with *D. immitis* mff were antigen-negative. The overall k concordance between the modified Knott’s test and the antigenic test was ≤0.2 (poor) (*p* = 0.000). The results of the logistic regression model showed a significant association between *Dirofilaria* exposure and the period of time the dogs had spent in the shelter at the time of sampling. Dogs housed in the shelter for 4 months (group 1) and between 4 months and 2 years (group 2) had higher *Dirofilaria* positivity than dogs in group 3 (housed for more than 2 years) (80.4% vs. 79.6% vs. 62.4%, respectively). Moreover, male dogs and older dogs (between 7 and 11 years of age) were more likely to be infected with *Dirofilaria* spp.

## 1. Introduction

Heartworm disease and subcutaneous dirofilariosis caused by *Dirofilaria immitis* and *D. repens* (Spirurida, Onchocercidae), respectively, are important vector-borne diseases, especially for dogs and cats (1–4). In addition, zoonotic infections, mostly due to *D. repens*, are a major public health problem (4). The epidemiological situation of canine dirofilariosis based on data reported in recent studies has shown that the prevalence of both *D. immitis* and *D. repens* is increasing in Europe and the southerneastern regions of Asia and Africa (1, 4, 5). However, there are studies that continuously report changes in the prevalence of both *D. immitis* and *D. repens* pathogens, identifying non-endemic areas with increased prevalence and previously endemic/hyper-endemic areas with decreasing prevalence (6–8). For example, the prevalence of *D. immitis* in northern Italy has decreased from >40% (9) to 8% in owned dogs over the last three decades (6, 7), due to the increased awareness of veterinary practitioners and better prevention strategies. In southern Italy, the number of autochthonous cases and foci of *D. immitis* in dogs has increased considerably (10, 11). This also occurs in the Campania region, particularly in coastal areas, where infections with *D. immitis* and *D. repens* have been reported more frequently and the prevalence of *D. repens* has increased from 2% (12) to 10% (13). As with *D. immitis*, hunting dogs, stray dogs, and dogs housed in kennels are more exposed, with seroprevalence ranging from 0.2 to 4.4% (14–16). The results of the questionnaire survey on heartworm and subcutaneous dirofilariosis addressed to veterinary practitioners throughout Italy (7) showed that at least one clinical case per year of cardiopulmonary dirofilariosis was diagnosed in dogs with frequent co-infestation by *D. immitis* and *D. repens* in Campania. Recent studies confirm the presence of autochthonous cases of *D. immitis* with a prevalence of approximately 0.1% (13, 17). Based on the last two reports of two cases of heartworm disease (HW) detected during post-mortem examination of two roaming dogs (17) and a screening study in which 10% of dogs tested positive for *D. repens* (13), both studies from the urban area of Castel Volturno in the Campania region of southern Italy, the aim of the present study was to describe an outbreak of infection in a dog shelter from the same area.

## 2. Materials and methods

**Figure 1:**
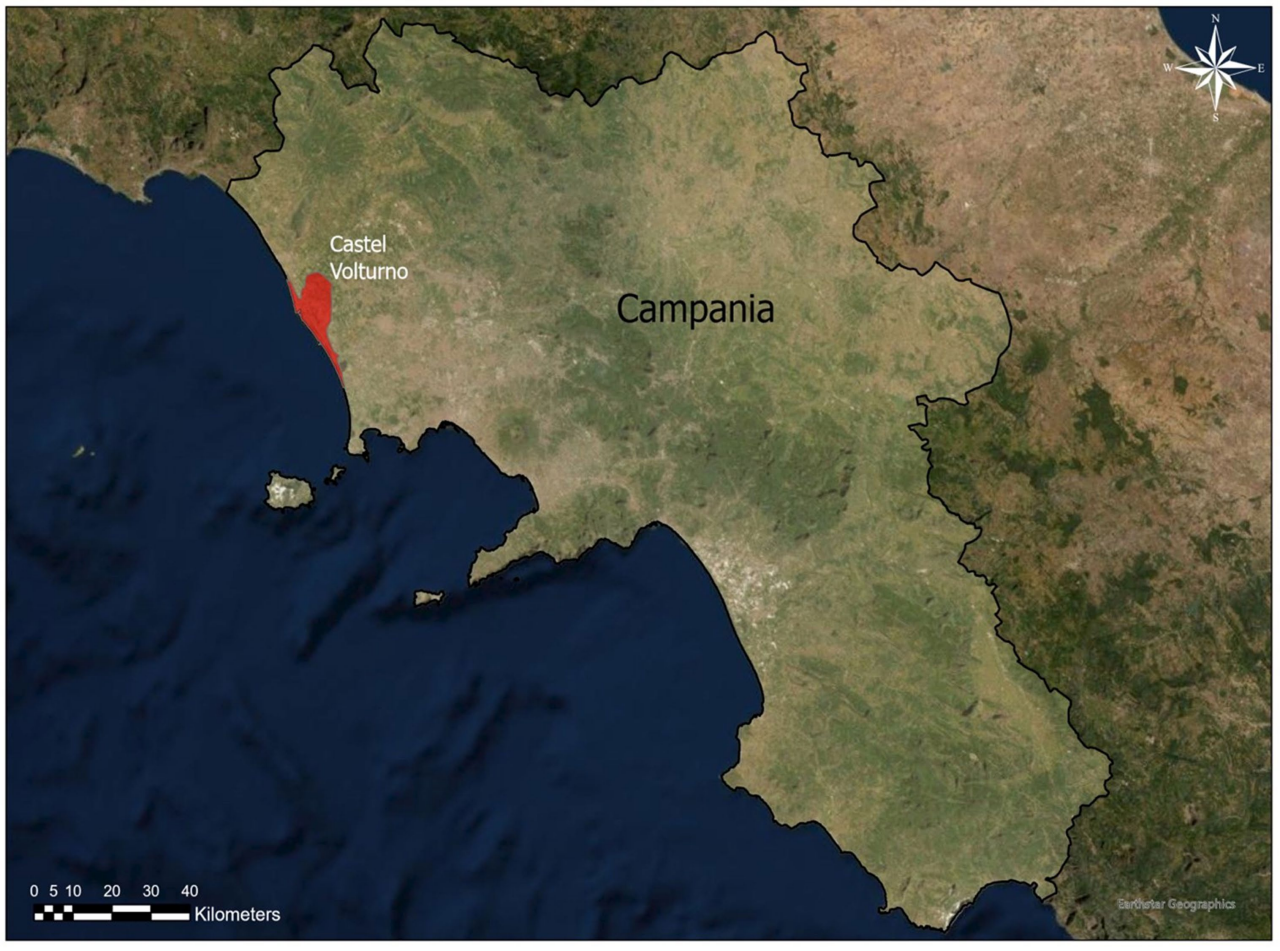
Shelter located in Castel Volturno, southern Italy.

The study was conducted in a dog shelter located in Castel Volturno (Figure 1), a deltaic coastal plain of the Volturno River, an area where veterinary practitioners have reported a high incidence of *D. repens* infection (13).

### 2.1. Ethics aspects

The study started in July 2020 and ended July 2021. The protocol of this study was approved by the Ethical Committee for Animal Experiments of the Department of Veterinary Medicine and Animal Production, University of Federico II Naples, Italy (Approval Number 0093204/2022). Informed consent was obtained from the shelter owner before starting the study.

### 2.2. Animals and collection of blood samples

Screening for *Dirofilaria* spp. was performed on all shelter dogs, with a few exceptions, as follows. Dogs younger than 9 months of age and dogs that have received doxycycline in the last month at the time of sampling, were excluded from the study. The shelter housed a total of 285 dogs, and based on the above exclusion criteria, a total of 260 dogs (96 females, 164 males, age range: 2–14 years) were selected and sampled. The dogs were housed in outdoor boxes, each box hosting 3–4 dogs. The management of the shelter in terms of parasitic disease diagnosis and treatment strategy was as follows: i) all dogs (new arrivals and others with symptoms) were tested for *Ehrlichia*/*Anaplasma* and *Leishmania infantum*; ii) all dogs were treated with a topical formulation (based on fipronil) against ectoparasites. Moreover, no screening for *Dirofilaria* spp., was performed until June 2020 (the start of the study) and no prophylactic treatment against *Dirofilaria* spp. was perfomed in the shelter. Anamnestic data were collected for the dogs studied, including age, sex, health status (i.e., activity level, appetite, health problems, skin abnormalities). Data were also collected on the length of stay of the dogs in the shelter at the time of sampling.

### 2.3. Laboratory analyses

All samples (*N* = 260 EDTA blood and serum samples) were examined for identification of circulating microfilariae (mff) using the modified Knott’s test (18) and for detection of *D. immitis* antigen using the Petcheck Canine Heartworm test (IDEXX). Moreover, molecular analyses [PCR protocol as described by Rishniw et al. (19)] were used to confirm the diagnosis of dirofilariosis in case of co-infections with both *D. immitis* and *D. repens*.

#### 2.3.1. Modified Knott test and molecular analysis

A modified Knott’s test was used for the detection of circulating mff of *Dirofilaria* spp. as follows. One mL of EDTA blood was mixed with 9 mL of distilled water and centrifuged at approximately 500 *g* for 3–5 min. The supernatant was removed from the tube and the content was stained with 1–2 drops of 1% methylene blue. One drop was placed on a microscope slide covered with a coverslip and observed under an optical microscope at 100X (18) to assess the level of microfilariaemia (mff/mL). The *Dirofilaria* negative/positive status of each dog included in the study was based on the examination of the entire contents of the sediment resulted from the modified Knott’s test. For molecular identification of microfilaria species, genomic DNA was extracted from 200 microliters of each blood sample using the DNeasy^®^ Blood and Tissue kit (Qiagen, Germany), according to the manufacturer’s instructions. Molecular analyses were performed according to the multiplex PCR protocol for simultaneous detection of the different *Dirofilaria* species, described by Rishniw et al. (19). Amplification of the internal transcribed spacer 2 (ITS2) region was carried out using the primers DIDR-F1 and DIDR-R1 with expected amplification product sizes of 542, 484, 578 and 584 bp for *D. immitis*, *D. repens*, *A. reconditum* and *A. dracunculoides*, respectively. The PCR reactions were increased to 25 μL of total volume, and included 5 μL of genomic DNA for each sample amplification, respectively (3).

### 2.4. Statistical analysis

The data of the dogs included in the study – i.e. gender, age, length of stay in the shelter at the time of sampling – were analysed by univariate statistical analysis using the Pearson’s Chi-square test for independence, to test the association with positivity for *D. immitis* and *D. repens*. For this purpose, dogs were divided into the following age classes: class 1) 1–3 years (*n* = 76); class 2) 4–6 years (*n* = 75); class 3) 7–11 years (*n* = 81); class 4) > 11 (*n* = 28) years. In addition, the dogs were divided into three groups based on the length of stay in the shelter at the time of sampling as follows: group 1) dogs that entered in the shelter in the last 4 months (*n* = 51); group 2) dogs that were housed in the shelter for more than 4 months up to 2 years (*n* = 108); group 3) dogs that were housed for more than 2 years (*n* = 109). The Kappa (k) statistic was used to measure the concordance between the antigenic test and the modified Knott’s test using the following criteria (20): ≤0.2 = poor; 0.21–0.40 = fair; 0.41–0.60 = moderate, 0.61–0.80 = good and ≥ 0.80 = very good. Therefore, all the dogs were divided into four groups based on the number of microfilariae/ml (0 mff = negative (group 1); 1–100 mff (group 2); 101–500 mff (group 3); 501 > mff (group 4). Moreover, multivariable (logistic regression) statistical analyses were performed using the *Dirofilaria* spp. exposure (positive/negative) as the dependent variable. Only the independent variables that showed significance (*p* < 0.01) in the univariate test were used for the logistic regression model. When an interaction between variables was suspected, the logistic regression model was run with and without these variables to assess possible effect modification on their behalf (21). The significance level was set at a *p* value <0.05. Data analysis was performed using SPSS version 17 software, Chicago, IL, United States.

## 3. Results

**Figure 2:**
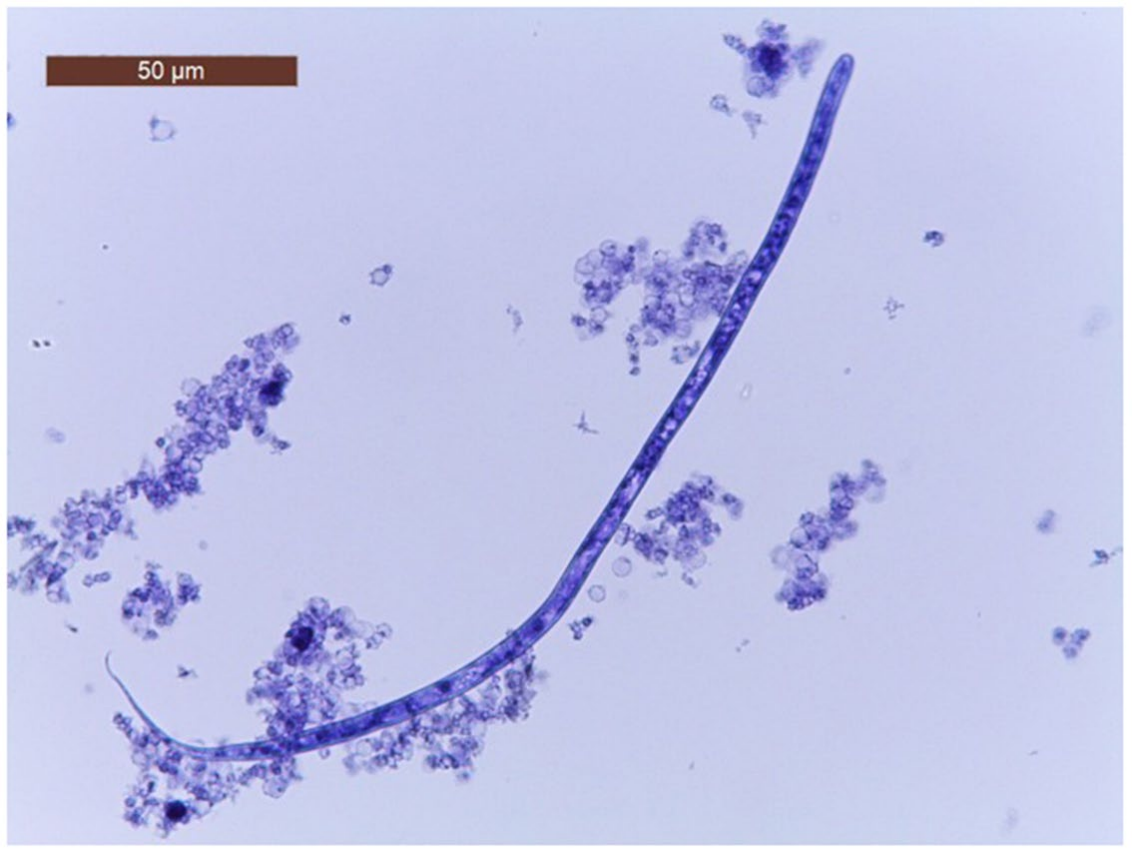
*Dirofilaria repens* mff, modified Knott test.

**Figure 3:**
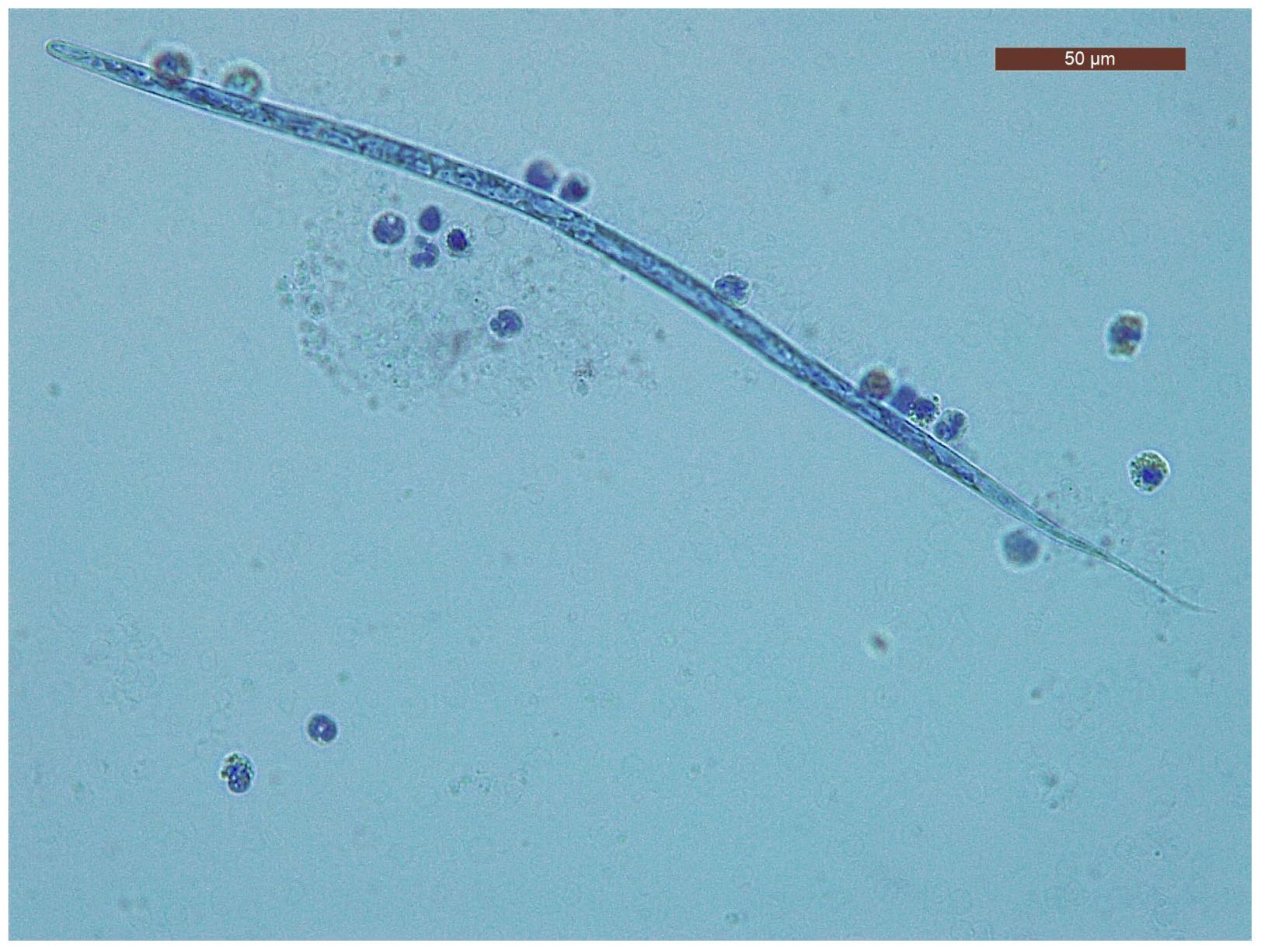
*Dirofilaria immitis* mff, modified Knott test.

The characteristics of the dogs from the shelter are shown in Table 1. The mean age of dogs in the trial was 7.06 years (minimum 1 year; maximum14 years). The modified Knott’s test revealed that 195/260 dogs (75%; 95% CI = 69.2–80.05) were positive for circulating mff of *Dirofilaria* spp. Specifically, 104/260 (40.5.%; 95% CI = 34.05.-46.25) dogs were positive for *D. immitis* mff (Figure 2) and 91/260 (35%; 95% CI = 29.3–41.17) were positive to *D. repens* mff (Figure 3). In addition, 72/260 (27.7%; 95% CI = 22.43–33.63) dogs had both *D. immitis* and *D. repens* mff. All dogs in which the modified Knott test showed coinfection with both pathogens were subsequently confirmed with a PCR test. Antigen testing showed 78/260 (30%; 95%CI = 24.57–36.03) dogs positive to *D. immitis* (Table 2). However, 26/104 (25%; 95%CI = 17.26–34.62) of the dogs with *D. immitis* mff were antigen-negative. Moreover, none of the positive dogs with *D. repens* mff mono-infection gave positive results on the antigen test for *D. immitis*.

**Table 1:** Positivity of *Dirofilaria* spp. in the dogs included in the study by category (length of stay in the shelter, age, gender).

Categories (total analysed *N* = 260)	*Dirofilaria* spp. (%/95%CI) Total positive *N* = 195
Length of stay in the shelter	
< 4 months (*n* = 51)	41 (80.4%) 95% CI = 66.45–89.71
> 4 months and < 2 years (*n* = 108)	86 (79.6%) 95% CI = 70.57–86.53
> 2 years (*n* = 109)	68 (62.4%) 95% CI = 52.55–71.33
*Age classes (N = 260)*	
(1)-1–3 years (*n* = 76)	49 (64.5%) 95% CI = 52.59–74.88
(2)-4–6 years (*n* = 75)	51 (68%) 95% CI = 55.10–78.04
(3)-7–11 years (*n* = 81)	70 (86.4%) 95% CI = 76.58–92.70
(4)- > 11 years (*n* = 28)	25 (89.3%) 95% CI = 70.63–97.19
*Gender*	
Females (*n* = 96)	54 (56.3%) 95% CI = 45.80–66.23
Males (*n* = 164)	141 (86%) 95% CI = 44.53–60.23

**Table 2:** Results of the modified Knott test, antigen test and PCR for all dogs included in the study.

Test	Di mono-inf.^a^	Dr mono-inf.^b^	Di + Dr^c^
Knott test	32/104 (30.8%)	19/91 (20.9%)	72/195 (36.9%)
Antigen test	78/260 (30.0%)	−	26/104 (25.0%)
PCR	−	−	+
Total a + c/b + c	104/260 (40.0%)	−	91/260 (35.0%)

Di mono-inf. = *D. immitis* mono-infection; Dr moni-inf. = *D. repens* mono-infection; “−”not performed; “+” performed.

The prevalence for both pathogens was almost twice as high in males (86%; 95%CI = 44.53–60.23) than in females (57%; 95% CI = 45.80–66.23) (Table 1). As expected, the prevalence was lowest in age class 1 (64.5%; 95% CI = 52 59–74.88) and highest in age classes 2 (68%; 95% CI = 55.10–78.04), 3 (86.4%; 95% CI = 76.58–92.70) and 4 (89.3%; 95% CI = 70.63–97.19), respectively. There was a significant difference with respect to the length of stay of the dogs in the shelter, reflecting mainly an increase in prevalence in group 1 (80.4%; 95% CI = 66.45–89.71 *p* = 0.012) and group 2 (86%; 95% CI = 70.57–86.53; *p* = 0.001) in which all dogs had newly arrived in the shelter for 4 months or between 4 months and 4 years, and came from different localities in the Campania region, including the city center of Naples. The results regarding the groups with the number of microfilariae were as follows: 0 mff/ml (group 1, *n* = 72 dogs); 1–100 mff/ml (group 2, *n* = 34 dogs); 101–500 mff/ml (group 3, *n* = 61 dogs); >501mff/ml (group 4, *n* = 93 dogs). The overall k concordance between the Knott’s test and the antigenic test was: ≤0.2 (=poor) (*p* = 0.000). The majority of the dogs had no health problems at the time of sampling. However, 13 dogs showed some symptoms: skin problems (skin lesions, poor quality of fur, itching), with five dogs showing small soft nodules in the subcutaneous tissues. All the dogs that presented nodules tested positive for *D. repens* in subsequent PCR testing. The results of the logistical regression model showed a significant association between *Dirofilaria* spp. exposure and length of stay in the shelter at the time of sampling. In this regard, the positivity to *Dirofilaria* was higher in dogs that had been housed in the shelter for 4 months (group 1) and in the dogs that had been housed between 4 months and 2 years (group 2), than in the other dogs of group 3 (dogs that had been housed for more than 2 years) (80.4%; OR = 1.93; 95% CI = 66.45–89.71; *p* = 0.012 and 79.6%; OR = 1.48; 95% CI = 70.57–86.53; *p* = 0.001) vs. (62.4%; 95% CI = 52.55–71.33; *p* = 0.089). In addition, male dogs (86%; OR = 2.02; value of *p* = 0.004) and older dogs (between 7 and 11 years: 86.4%; OR = 1.65; *p* = 0.003 and more than 11 years: 89.3%; OR = 3.83; *p* = 0.002) were more exposed to *Dirofilaria* infection. Moreover, all dogs were previously tested for *Leishmania* (before this study) and none of them resulted positive. However, to exclude possible cross-reactions with *Angiostrongylus vasorum* to the antigenic test of *D. immitis,* which has been reported (22), all dogs in this study were tested for *A. vasorum* antigen, but the result was negative.

## 4. Discussion and conclusions

The data presented in this manuscript on the occurrence of *Dirofilaria* spp. in a shelter dog from southern Italy (Campania region) are consistent with those endemic/hyperendemic areas in the Mediterranean region (4, 8, 10, 11, 23–25). The present study revealed a high prevalence of both *D. immitis* (40%) and *D. repens* (35%) in a dog shelter located in a semi-urban area of Castel Volturno, representing a high risk of infection not only for companion animals, but also for humans. Although the screening included only one dog shelter in the Campania region, the survey of dirofilariosis status was conducted for the entire dog population housed in the shelter. In addition, two previous studies conducted in the same area – one reporting two cases of HW disease at post-mortem examination of two roaming dogs, and the second showing the presence of both *D. immitis* and *D. repens* in owned dogs (13, 17) – had underscored the need for further evaluation of dirofilariosis risk in dogs in the coastal area of Castel Volturno, which provides suitable climatic and environmental conditions to sustain year-round populations of mosquito vectors and *Dirofilaria* pathogens (13, 26).

The overall prevalence of *D. immitis* and *D. repens* (microfilariae) in the studied dog population in the Campania region was 75%. This result may be indicative of an increased risk of dirofilariosis in the studied area, although further studies with larger dog populations (both stray and owned dogs), should be investigated for *Dirofilaria* spp. However, this study is consistent with other studies recently conducted in other regions of southern Italy, where a high prevalence of *D. immitis* has been reported [e.g., (10, 11, 27–29)].

The apparent emerging risk of *D. immitis* infection in the Campania region raises important questions. There are many factors related to the vectors (presence of competent mosquito species) and the hosts (owner compliance with chemoprophylaxis, presence of wild canid reservoirs) that may influence the prevalence of HW disease in different areas of southern Italy. There are few studies in the literature on the prevalence of *D. immitis* in the Campania region that showed an increase in seroprevalence in dogs over time (11, 12, 14, 30).

*Dirofilaria repens* is considered one of the most widespread zoonotic pathogens in Europe, and the occurrence of zoonotic cases goes hand in hand with infection in dogs. Currently, *D. repens* infection occurs in southern Italy, especially in coastal areas. The results of the questionnaire survey on dirofilariosis addressed to freelance veterinarians throughout Italy (7) showed clinical cases of *D. repens* and/or *D. immitis* in dogs in the Campania region. The prevalence of *D. repens* ranges from 1.5 to 10% in Campania (13, 17) and 0.8% in the neighboring Molise region (31). Our study showed prevalence values of *D. repens* (35%) higher than those reported in the above studies. However, sheltered dogs are generally more exposed to canine vector-borne diseases (32, 33), and therefore future investigations will need to be conducted on owned dogs in the area to better understand the epidemiology of *Dirofilaria* spp.

Given the zoonotic potential of both *D. repens* and *D. immitis*, vector control and systematic treatment of domestic and stray dogs should be considered to reduce the risk of human infection. Humans are usually dead-end hosts, although there have been cases with fertile adults and/or microfilariaemia due to *D. repens* (34–37). In most patients, *D. immitis* locates in the lungs and causes pulmonary dirofilariosis, whereas *D. repens* appears as nodules in subcutaneous tissues or as free worms under the conjunctiva (ocular dirofilariosis). Pulmonary dirofilariosis may be confused with pulmonary neoplasms (benign, carcinoma, metastasis), tuberculosis, or mycotic infections (37) and presents a diagnostic challenge.

The lack of entomological surveys in the Campania region is an important gap in the knowledge of *Dirofilaria* transmission of in this coastal area of southern Italy, which has several favorable environmental characteristics for the survival and development of the vector and, consequently, for the spread of both *D. immitis* and *D. repens* (26).

In the present study, almost half of the positive dogs had been infected before their arrival at the shelter. In addition, statistical analysis showed that male dogs were more exposed to *D. immitis* than females. To date, there is no evidence of an actual predisposition of sex to these pathogens, although many studies have reported similar results (38–40). On the other hand, age is considered an important risk factor for *D. immitis* infection. In fact, the prevalence in older dogs is generally higher than in younger dogs, which is related to the increasing duration of exposure to mosquitoes (41, 42). Accordingly, our study showed the highest prevalence in dogs in the age classes >11 years (89.3%) and 7–11 years (86.4%) and the lowest in dogs in the age classes 1–3 years (64.5%) and 4–6 years (68%).

From a diagnostic point of view, our results showed that the antigen test alone (which is usually the most commonly used for the diagnosis of *D. immitis*) was not sufficient, as the modified Knott test detected a higher number of positive samples. This finding is not consistent with other studies (43, 44), in which the proportion of antigen-positive dogs was higher than that of Knott-positive dogs. However, one study showed the same pattern with dogs resulted positive with the modified Knott test but negative with the antigen test (45). The disagreement between the modified Knott test and the antigen test in the present study could be due to the low concentration of free antigen available for detection by serological tests. Also, it has been reported that the immune-complex formation can lead to false-negative antigen test results and that pre-heating serum samples can disrupt immune-complexes, leading to a positive test result (28, 46, 47). However, the authors chose not to pre-heat the serum samples because there were also dogs co-infected with *D. repens*. Moreover, it has been previously reported that mono-infection with *D. repens* can lead to false-positive antigen tests for *D. immitis* (48, 49). In addition, blocked antigens are more likely to occur in microfilaraemic dogs, in which a higher antibody response occurs compared to amicrofilaremic dogs (43, 50); this may also justify our results from the present study.

In this study, the antigen of *D. immitis* was detected using only one test (Petchek HW antigen test). However, as reported in a previous study by Constantinoiu et al. (43), double checking of antigen-negative samples from dogs with mff of *D. immitis*, using a different antigen test might be beneficial for accurate diagnosis of HW disease. Several studies have demonstrated that molecular methods can be a highly sensitive and specific analytical tool for simultaneous diagnosis and characterization of infections, and provide more reliable data compared to serological and parasitological methods (3, 51). In the present study, there were no differences between the modified Knott test and molecular tests. Similar results were obtained in studies in which the modified Knott test was found to be effective, sensitive, and compatible with PCR (3, 52).

In conclusion, HW disease is spreading over time for the reasons mentioned above, and southern Italy, once considered a low-risk area, is increasingly becoming the site of autochthonous outbreaks. We conclude that dog shelters in southern Italy are hotspots for *Dirofilaria* spp. transmission and strongly recommend education and veterinary advice for systematic treatment and the use of a diagnostic strategy with multiple tests to detect positive dogs in a given population (10, 11, 33). Proper management of these infections should be based on an effective and correct approach to diagnosis and up-to-date therapy, as well as practical prophylactic measures to protect animals and humans.

## Data availability statement

The raw data supporting the conclusions of this article will be made available by the authors, without undue reservation.

## Ethics statement

The animal study was reviewed and approved by the ethical committee of animal experiments of the Department of Veterinary Medicine and Animal Production, University of Federico II Naples, Italy (Approval Number 0093204/2022). Written informed consent was obtained from the owners for the participation of their animals in this study.

## Author contributions

LC, VC, AB, and LR: conceptualization. LC, VC, SI, and AB: data curation. LC, VC, and SI: formal analysis. LC and VC: investigation. LC and AB: methodology. LC, LR, and MM: project administration. LR and MM: supervision. LC, VC, and AB: writing – original draft. LR and MM: writing – review & editing. All authors contributed to the article and approved the submitted version.

## Conflict of interest

The authors declare that the research was conducted in the absence of any commercial or financial relationships that could be construed as a potential conflict of interest.

## Publisher’s note

All claims expressed in this article are solely those of the authors and do not necessarily represent those of their affiliated organizations, or those of the publisher, the editors and the reviewers. Any product that may be evaluated in this article, or claim that may be made by its manufacturer, is not guaranteed or endorsed by the publisher.

## References

[1] Capelli G Genchi C Baneth G Bourdeau P Brianti E Cardoso L. Recent advances on *Dirofilaria repens* in dogs and humans in Europe. Parasites &Vectors. (2018) 11:663. doi: 10.1186/s13071-018-3205-x 30567586 PMC6299983

[2] Ciuca L Roman C Prisco F Miron L Acatrinei D Paciello O. First report of *Dirofilaria repens* infection in a microfilaraemic cat from Romania. Vet Parasitol Reg Stud. (2020) 22:100497. doi: 10.1016/j.vprsr.2020.100497 33308740

[3] Ciuca L Vismarra A Lebon W Beugnet F Morchon R Rinaldi L. New insights into the biology, diagnosis and immune response to *Dirofilaria repens* in the canine host. Vet Parasitol X. (2020) 4:100029. doi: 10.1016/j.vpoa.2020.100029 32904796 PMC7458378

[4] Genchi C Kramer LH. The prevalence of *Dirofilaria immitis* and *D. repens* in the Old World. Vet Parasitol. (2020) 280:108995. doi: 10.1016/j.vetpar.2019.108995 32155518

[5] McCall JV Genchi C Kramer LH Guerrero J Venco L. Heartworm disease in animals and humans. Adv Parasitol. (2008) 66:193–285. doi: 10.1016/S0065-308X(08)00204-2 18486691

[6] Genchi C Rinaldi L Mortarino M Genchi M Cringoli G. Climate and dirofilaria infection in Europe. Vet Parasitol. (2009) 163:286–92. doi: 10.1016/j.vetpar.2009.03.026, PMID: 19398159

[7] Genchi M Rinaldi L Venco L Cringoli G Vismarra A Kramer L. *Dirofilaria immitis* and *D. repens* in dog and cat: a questionnaire study in Italy. Vet Parasitol. (2019) 267:26–31. doi: 10.1016/j.vetpar.2019.01.014, PMID: 30878081

[8] Montoya-Alonso JA Morchon R Falcon-Cordon Y Falcon-Cordon S Simon F Carreton E. Prevalence of heartworm in dogs and cats of Madrid, Spain. Parasites Vectors. (2017) 10:354. doi: 10.1186/s13071-017-2299-x 28747221 PMC5530495

[9] Guerrero J Genchi C Vezzoni A Ducos De Lahitte J Bussieras J Rojo FA. (1989). Distribution of *Dirofilaria immitis* in selected areas of Europe and South America. Otto GF (Ed.), Proceedings of the Heartworm Symposium’ in Southern Italy. Animals (Basel) 11

[10] Brianti E Panarese R Napoli E De Benedetto G Gaglio G Bezerra-Santos MA. *Dirofilaria immitis* infection in the Pelagie archipelago: the southernmost hyperendemic focus in Europe. Transbound Emerg Dis. (2022) 69:1274–80. doi: 10.1111/tbed.14089, PMID: 33787005

[11] Napoli E De Benedetto G Ciuca L Bosco A Lia RP Veneziano V. New distribution patterns of *Dirofilaria immitis* in Italy. Front Vet Sci. (2023) 10:1162403. doi: 10.3389/fvets.2023.1162403 37215465 PMC10193386

[12] Cringoli G Rinaldi L Veneziano V Capelli G. A prevalence survey and risk analysis of filariosis in dogs from the Mt. Vesuvius area of southern Italy. Vet Parasitol. (2001) 102:243–52. doi: 10.1016/S0304-4017(01)00529-5, PMID: 11777604

[13] Ferrara M Maglione R Ciccarelli D Mundis SJ Di Loria A Pisaroglo de Carvalho M. Prevalence of *Dirofilaria repens* in dogs living in deltaic coastal plain of the Volturno River (Italy): a geographical risk model of infection. J Helminthol. (2022) 96:e12. doi: 10.1017/S0022149X22000062, PMID: 35195063

[14] Del Prete L Maurelli MP Pennacchio S Bosco A Musella V Cringoli CL. *Dirofilaria immitis* and Angiostrongylus vasorum: the contemporaneous detection in kennels. BMC Vet Res. (2015) 11:305. doi: 10.1186/s12917-015-0619-y 26689960 PMC4687387

[15] Petruccelli A Ferrara G Iovane G Schettini R Ciarcia R Caputo V. Seroprevalence of *Ehrlichia* spp., Anaplasma spp., *Borrelia burgdorferi sensu lato*, and *Dirofilaria immitis* in Stray Dogs, from 2016 to 2019, in Southern Italy. Animals (Basel). (2020) 11:9. doi: 10.3390/ani11010009 33374634 PMC7822448

[16] Piantedosi D Neola B D'Alessio N Di Prisco F Santoro M Pacifico L. Seroprevalence and risk factors associated with *Ehrlichia canis*, Anaplasma spp., *Borrelia burgdorferi* sensu lato, and *D. immitis* in hunting dogs from southern Italy. Parasitol Res. (2017) 116:2651–60. doi: 10.1007/s00436-017-5574-z, PMID: 28776227

[17] Santoro M Miletti G Vangone L Spadari L Reccia S Fusco G. Heartworm disease (*Dirofilaria immitis*) in two roaming dogs from the urban area of Castel Volturno Southern Italy. Front Vet Sci. (2019) 28:270. doi: 10.3389/fvets.2019.00270 PMC6722179 31555670

[18] Genchi M Ciuca L Vismarra A Ciccone E Cringoli G Kramer L. Evaluation of alternative reagents on the performance of the modified Knott's test. Vet Parasitol. (2021) 298:109555. doi: 10.1016/j.vetpar.2021.109555, PMID: 34425345

[19] Rishniw M Barr SC Simpson KW Frongillo MF Franz M Dominguez Alpizar JL. Discrimination between six species of canine microfilariae by a single polymerase chain reaction. Vet Parasitol. (2006) 135:303–14. doi: 10.1016/j.vetpar.2005.10.013, PMID: 16289566

[20] Altman DG, (1991) Practical Statistics for Medical Research. Chapman & Hall; Boca Raton (1991). pp. 277–321.

[21] Hosmer DW Lemeshow S. Applied Logistic Regression. 2nd ed. New York: John Wiley & Sons, Inc. (2000).

[22] Schnyder M Deplazes P. Cross-reactions of sera from dogs infected with Angiostrongylus vasorum in commercially available *Dirofilaria immitis* test kits. Parasit Vectors. (2012) 5:258. doi: 10.1186/1756-3305-5-258 23148786 PMC3503614

[23] Alho AM Meireles J Schnyder M Cardoso L Belo S Deplazes P. *Dirofilaria immitis* and Angiostrongylus vasorum: the current situation of two major canine heartworms in Portugal. Vet Parasitol. (2018) 252:120–6. doi: 10.1016/j.vetpar.2018.01.008, PMID: 29559132

[24] Genchi C Bowman D Drake J. Canine heartworm disease (*Dirofilaria immitis*) in Western Europe: survey of veterinary awareness and perceptions. Parasit Vectors. (2014) 7:206. doi: 10.1186/1756-3305-7-206 24779376 PMC4013803

[25] Vrhovec MG Pantchev N Failing K Bauer C Travers-Martin N Zahner H. Retrospective analysis of aanine vector-borne diseases (CVBD) in Germany with emphasis on the endemicity and risk factors of leishmaniosis. Parasitol Res. (2017) 116:131–44. doi: 10.1007/s00436-017-5499-6, PMID: 28717956

[26] Genchi C Rinaldi L Cascone C Mortarino M Cringoli G. Is heartworm disease really spreading in Europe? Vet Parasitol. (2005) 133:137–48. doi: 10.1016/j.vetpar.2005.04.009, PMID: 15885913

[27] Mendoza-Roldan J Benelli G Panarese R Iatta R Furlanello T Beugnet F. Leishmania infantum and *Dirofilaria immitis* infections in Italy, 2009-2019: changing distribution patterns. Parasit Vectors. (2020) 13:193. doi: 10.1186/s13071-020-04063-9 32293524 PMC7161282

[28] Panarese R Iatta R Mendoza-Roldan JA Szlosek D Braff J Liu J. Comparison of diagnostic tools for the detection of *Dirofilaria immitis* infection in dogs. Pathogens. (2020) 9:499. doi: 10.3390/pathogens9060499 32580453 PMC7350293

[29] Panarese R Iatta R Beugnet F Otranto D. Incidence of *Dirofilaria immitis* and Leishmania infantum infections in sheltered dogs from southern Italy. Transbound Emerg Dis. (2022) 69:891–4. doi: 10.1111/tbed.14025, PMID: 33547868

[30] Rinaldi L Del Prete L Noviello E Musella V Cringoli G. (2015). Dirofilaria infection in dogs from the Campania region of southern Italy. Second conference on neglected vectors and vector-borne diseases (EurNegVec) with management and working group meetings on the COST ACTION TD1303, Izmir, Turkey, March 31- April 2, 2015, 80.

[31] Gizzarelli M Foglia Manzillo V Ciuca L Morgoglione ME Fayala NEIHB Cringoli G. Simultaneous detection of parasitic vector borne diseases: a robust cross-sectional survey in hunting, stray and sheep dogs in a Mediterranean area. Front Vet Sci. (2019) 6:288. doi: 10.3389/fvets.2019.00288 31555672 PMC6727173

[32] Lane JN Litster A Little SE Rodriguez JY Mwacalimba KK Sundstrom KD. Optimizing heartworm diagnosis in dogs using multiple test combinations. Parasit Vectors. (2021, 224) 14. doi: 10.1186/s13071-021-04715-4 PMC8074442 33902687

[33] Otranto D Dantas-Torres F Mihalca AD Traub RJ Lappin M Baneth G. Zoonotic parasites of sheltered and stray dogs in the era of the global economic and political crisis. Trends Parasitol. (2017) 33:813–25. doi: 10.1016/j.pt.2017.05.013, PMID: 28648798

[34] Bronshteyn AM Malyshev NA Jarov SN Fedianina LV Frolova AA Supriaga VG. A first autochthonous human case of the long standing microfilaraemia due to *Dirofilaria repens* in Russia and a first experience of combined therapy of dirofilariasis repens. Epidemiol Infect Dis. (2013) 3:47–52. doi: 10.17816/EID40741

[35] Grandi G Morchon R Kramer L Kartashev V Simon F. Wolbachia in *Dirofilaria repens*, an agent causing human subcutaneous Dirofilariasis. J Parasitol. (2008) 94:1421–3. doi: 10.1645/GE-1575.1, PMID: 19127968

[36] Poppert S Hodapp M Krueger A Hegasy G Niesen WD Kern WV. *Dirofilaria repens* infection and concomitant meningoencephalitis. Emerg Infect Dis. (2009) 15:1844–6. doi: 10.3201/eid1511.090936, PMID: 19891881 PMC2857255

[37] Simón F González-Miguel J Diosdado A Gómez PJ Morchón R Kartashev V. The complexity of zoonotic Filariasis Episystem and its consequences: a multidisciplinary view. Biomed Res Int. (2017) 2017:6436130. doi: 10.1155/2017/6436130, PMID: 28642878 PMC5469992

[38] Mircean V Dumitrache MO Gyorke A Pantchev N Jodies R Mihalca AD. Seroprevalence and geographic distribution of *Dirofilaria immitis* and tick-borne infections (*Anaplasma phagocytophilum*, *Borrelia burgdorferi* sensu lato, and *Ehrlichia canis*) in dogs from Romania. Vector Borne Zoonotic Disease. (2012) 12:595–604. doi: 10.1089/vbz.2011.0915, PMID: 22607068

[39] Traversa D Aste G Milillo P Capelli G Pampurini F Tunesi C. Autochthonus foci of canine and feline infections by *Dirofilaria immitis* and *Dirofilaria repens* in Central Italy. Vet Parasitol. (2010) 169:128–32. doi: 10.1016/j.vetpar.2009.12.034, PMID: 20097479

[40] Yildirim A Ica A Atalay O Duzlu O Inci A. Prevalence and epidemiological aspects of *Dirofilaria immitis* in dogs from Kayseri Province, Turkey. Res Vet Science. (2007) 82:358–63. doi: 10.1016/j.rvsc.2006.08.006, PMID: 17064741

[41] Vieira AL Vieira MJ Oliveira JM Simões AR Diez-Baños P Gestal J. Prevalence of canine heartworm (*Dirofilaria immitis*) disease in dogs of Central Portugal. Parasite. (2014) 21:5. doi: 10.1051/parasite/2014003, PMID: 24534524 PMC3927308

[42] Yuasa Y Hsu TH Chou CC Huang CC Huang WC Chang CC. The comparison of spatial variation and risk factors between mosquito-borne and tick-borne diseases: seroepidemiology of *Ehrlichia canis*, Anaplasma spp. and *Dirofilaria immitis* in dogs. Comp Immunol Microbiol Infect Dis. (2012) 35:599–606. doi: 10.1016/j.cimid.2012.08.001 22925931

[43] Constantinoiu C Croton C Paterson MBA Knott L Henning J Mallyon J. Prevalence of canine heartworm infection in Queensland, Australia: comparison of diagnostic methods and investigation of factors associated with reduction in antigen detection. Parasit Vectors 10. (2023) 16:63. doi: 10.1186/s13071-022-05633-9 36765417 PMC9921331

[44] Panarese R Iatta R Latrofa MS Zatelli A Ćupina AI Montarsi F. Hyperendemic *Dirofilaria immitis* infection in a sheltered dog population: an expanding threat in the Mediterranean region. Int J Parasitol. (2020) 50:555–9. doi: 10.1016/j.ijpara.2020.04.002 32479831

[45] Ciuca L Vismarra A Constanza D Di Loria A Meomartino L Ciaramella P. Efficacy of oral, topical and extended-release injectable formulations of moxidectin combined with doxycycline in Dirofilaria immitis naturally infected dogs. Parasit Vectors 6. (2023) 16:54. doi: 10.1186/s13071-023-05673-9 36740705 PMC9901089

[46] Ciuca L Genchi M Kramer L Mangia C Miron LD Prete LD. Heat treatment of serum samples from stray dogs naturally exposed to Dirofilaria immitis and *Dirofilaria repens* in Romania. Vet Parasitol. (2016) 225:81–5. doi: 10.1016/j.vetpar.2016.05.032, PMID: 27369579

[47] Drake J Gruntmeir J Merritt H Allen L Little SE. False negative antigen tests in dogs infected with heartworm and placed on macrocyclic lactone preventives. Parasit Vectors. (2015) 8:68. doi: 10.1186/s13071-015-0698-4 25648086 PMC4336501

[48] Sobotyk C Savadelis MD Verocai GG. Detection and cross-reaction of *Dirofilaria repens* using a commercial heartworm antigen test kit. Vet Parasitol. (2021) 289:109302. doi: 10.1016/j.vetpar.2020.109302, PMID: 33352522

[49] Venco L Manzocchi S Genchi M Kramer LH. Heat treatment and false-positive heartworm antigen testing in ex vivo parasites and dogs naturally infected by *Dirofilaria repens* and Angiostrongylus vasorum. Parasit Vectors Nov. (2017) 9:476. doi: 10.1186/s13071-017-2444-6 PMC5688472 29143662

[50] Morchon R Lopez-Belmonte J Bazzocchi C Grandi G Kramer L Simon F. Dogs with patent *Dirofilaria immitis* infection have higher expression of circulating IL-4, IL-10 and iNOS mRNA than those with occult infection. Vet Immunol Immunopathol. (2007) 115:184–8. doi: 10.1016/j.vetimm.2006.10.004, PMID: 17112598

[51] Little S Saleh M Wohltjen M Nagamori Y. Prime detection of *Dirofilaria immitis*: understanding the influence of blocked antigen on heartworm test performance Parasit. Vectors. (2018) 11:186. doi: 10.1186/s13071-018-2736-5 PMC5859648 29554955

[52] Gomes-de-Sá S Santos-Silva S Moreira AS Barradas PF Amorim I Cardoso L. Assessment of the circulation of *Dirofilaria immitis* in dogs from northern Portugal through combined analysis of antigens, DNA and parasite forms in blood. Acta Trop. (2023) 239:106799. doi: 10.1016/j.actatropica.2022.106799, PMID: 36572345

