# Corrigendum: Emerging risk of *Dirofilaria* spp. infection in shelter dogs in southern Italy

**DOI:** 10.3389/fvets.2023.1290005

**Published:** 2023-09-27

**Authors:** Lavinia Ciuca, Valeria Caruso, Sergio Illiano, Antonio Bosco, Maria Paola Maurelli, Laura Rinaldi

**Affiliations:** Department of Veterinary Medicine and Animal Production, Unit of Parasitology, University of Federico II, Naples, Italy

**Keywords:** *Dirofilaria immitis*, *Dirofilaria repens*, southern Italy, shelter dogs, zoonotic risk

In the published article, there was an error in the legend for Figure 2 as published:

*Dirofilaria immitis* mff, modified Knott test

The corrected legend appears below.

Figure 2. *Dirofilaria repens* mff, modified Knott test.

In the published article, there was an error in the legend for Figure 3 as published: *Dirofilaria repens* mff, modified Knott test.

The corrected legend appears below.

Figure 3. *Dirofilaria immitis* mff, modified Knott test.

The authors apologize for this error and state that this does not change the scientific conclusions of the article in any way. The original article has been updated.

